# Sheffer operation in relational systems

**DOI:** 10.1007/s00500-021-06466-x

**Published:** 2021-11-17

**Authors:** Ivan Chajda, Helmut Länger

**Affiliations:** 1 Faculty of Science, Department of Algebra and Geometry, Palacký University Olomouc, 17. listopadu 12, 771 46 Olomouc, Czech Republic; 2 TU Wien, Faculty of Mathematics and Geoinformation, Institute of Discrete Mathematics and Geometry, Wiedner Hauptstraße 8-10, 1040 Vienna, Austria

**Keywords:** Relational system, Directed relational system, Involution, Sheffer operation, Sheffer groupoid, Twist product, Kleene relational system

## Abstract

The concept of a Sheffer operation known for Boolean algebras and orthomodular lattices is extended to arbitrary directed relational systems with involution. It is proved that to every such relational system, there can be assigned a Sheffer groupoid and also, conversely, every Sheffer groupoid induces a directed relational system with involution. Hence, investigations of these relational systems can be transformed to the treatment of special groupoids which form a variety of algebras. If the Sheffer operation is also commutative, then the induced binary relation is antisymmetric. Moreover, commutative Sheffer groupoids form a congruence distributive variety. We characterize symmetry, antisymmetry and transitivity of binary relations by identities and quasi-identities satisfied by an assigned Sheffer operation. The concepts of twist products of relational systems and of Kleene relational systems are introduced. We prove that every directed relational system can be embedded into a directed relational system with involution via the twist product construction. If the relation in question is even transitive, then the directed relational system can be embedded into a Kleene relational system. Any Sheffer operation assigned to a directed relational system ${\mathbf {A}}$ with involution induces a Sheffer operation assigned to the twist product of ${\mathbf {A}}$.

Introduction

Relational systems form one of the most general mathematical structures. Almost all structures appearing in algebra can be considered as relational structures. Such structures were studied for a long lime, see the pioneering work by Riguet (1948) containing elementary properties and constructions with binary relations and the paper by Fraissé (1954). On the other hand, in contrast to publications in algebra, not so many of papers are devoted to relational systems. One of the reasons is that there are not so powerful tools for investigating relations as there are for algebras. This is also the reason why relational systems do not appear so often in applications both in mathematics and outside. One important application of relational systems are, e.g., Kripke systems used in the formalization of several non-classical logical systems.

The authors introduced formerly several methods where relational systems are connected with various accompanying algebras, and hence their properties can be transformed into algebraic language, and the problems are solved by tools developed in general algebra. Let us mention, e.g., Chajda and Länger (2013) and Chajda and Länger (2016a) where certain groupoids similar to directoids are assigned to a relational system. In Chajda and Länger (2016b) and Chajda et al. (2015), this approach is applied to relational systems equipped with a unary operation. For ternary relations, such an approach was used in Chajda et al. (2013). In Bonzio and Chajda (2018), relational systems are treated similarly as residuated posets. This is important because residuated posets serve as an algebraic semantics of a certain kind of substructural logic, see (Chajda and Länger 2021), and hence also the considered relational systems can play a similar role in a more general setting.

In the present paper, we extend this list of used tools by the so-called Sheffer operation. Remember that the Sheffer operation introduced by Sheffer (1913) was used in Boolean algebras as a very successful tool since this operation can replace all other Boolean operations. Namely, every Boolean operation, both basic or derived, can be expressed by repeatedly using the Sheffer operation, see e.g., (Birkhoff 1979). In today terminology, the clone of Boolean functions is generated by the Sheffer operation. This has a surprising and very successful application in technology because in switching circles in particular in computer processors, it suffices to use only one binary operation, namely the Sheffer one. Then the technology of production of such chips is much easier and cheaper than it was in the beginning of computer era when several parts of the computer were composed by at least two different kinds of diodes (e.g., one for conjunction and the other one for negation). As it was shown by the first author in Chajda (2005), a Sheffer operation can be introduced not only in Boolean algebras but also in orthomodular lattices or even in ortholattices (see Birkhoff 1979 for these concepts). These algebras form an algebraic semantics of the logic of quantum mechanics, see e.g., Birkhoff and von Neumann (1936) or Husimi (1937). However, it turns out that such lattices may not model this propositional calculus precisely, see e.g., (Finch 1970). The reason is that in these logics, disjunction does not necessarily exist for all elements, i.e., that supremum of two elements need not exist if these elements are not orthogonal, see Chajda and Kolařík (2014) and Finch (1970). Hence, the so-called orthomodular posets and orthoposets were introduced. This was the reason why the concept of Sheffer operation was transferred from ortholattices to orthomodular posets and orthoposets, or, more generally, to posets with an involution or a complementation, see Chajda and Kolařík (2021).

The next natural step is to extend this method from posets to more general relational systems. In order to avoid difficulties with not everywhere defined operations and some other drawbacks, we consider so-called directed relational systems where the relation is reflexive and equipped with a unary involution operation. The authors show that also in this case, a kind of Sheffer operation can be introduced and the corresponding groupoid characterizes the given relational system. At first, we show that similarly as for Boolean algebras, using an assigned Sheffer operation, we can conversely recover not only the involution, but also the given binary relation.

The goals and benefits of our approach are as follows:

- At first, we show that similarly as for Boolean algebras where all the operations can be recovered by means of the Sheffer operation, also here the unary operation and the given binary relation can be reconstructed by means of only one specific Sheffer operation.
- We show that some basic properties of binary relations can be characterized by means of identities and quasi-identities in this Sheffer operation and hence one can use a purely algebraic approach to these relational systems.
- Since the class of Sheffer groupoids assigned to the class of relational systems with antitone involution forms a variety, also some important congruence properties can be investigated for relational systems.
- We describe connections between homomorphisms of Sheffer groupoids and homomorphisms of assigned relational systems.
- We derive Kleene relational systems by using the twist product construction and introduce a Sheffer operation on them in order to be able to apply the above-mentioned tools and results.

## Basic concepts

The Sheffer operation was introduced by Sheffer (1913) in Boolean algebras. If ${\mathbf {B}}=(B,\vee ,\wedge ,{}',0,1)$ is a Boolean algebra and one defines

$$\begin{aligned} x|y:=x'\vee y', \end{aligned}$$

then | is just the Sheffer operation on ${\mathbf {B}}$. At first, we introduce the concept of Sheffer operation in a general setting as follows.

In the following, the notation $t_1(x_1,\ldots ,x_n)\approx t_2(x_1,\ldots ,x_n)$ means that $t_1(x_1,\ldots ,x_n)=t_2(x_1,\ldots ,x_n)$ holds for all $x_1,\ldots ,x_n$ of the corresponding base set.

### Definition 2.1

A *Sheffer operation* on a non-void set *A* is a binary operation | on *A* satisfying the following identities:

1 $$\begin{aligned}&(x|y)|(x|x)\approx x, \end{aligned}$$

2 $$\begin{aligned}&(x|y)|(y|y)\approx y. \end{aligned}$$

A *groupoid* is an algebra of type (2). A *Sheffer groupoid* is a groupoid (*A*, |) where | is a Sheffer operation on *A*.

Hence, the class of Sheffer groupoids is determined by identities, i.e., it forms a variety.

### Example 2.2

If $A:=\{a,b,c,d\}$ and the binary operation | on *A* is defined by

$$\begin{aligned} \begin{array}{c|cccc} | &{}\quad a &{}\quad b &{}\quad c &{}\quad d \\ \hline a &{}\quad a &{}\quad c &{}\quad d &{}\quad c \\ b &{}\quad c &{}\quad b &{}\quad d &{}\quad c \\ c &{}\quad a &{}\quad b &{}\quad d &{}\quad c \\ d &{}\quad a &{}\quad b &{}\quad d &{}\quad c, \end{array} \end{aligned}$$

then (*A*, |) is a Sheffer groupoid.

It is worth noticing that the Sheffer operation in a Boolean algebra satisfies the identities (1) and (2), and hence our new concept is sound.

An *antitone involution* on a lattice $(L,\vee ,\wedge )$ is a unary operation $'$ on *L* satisfying

(i) $$x''\approx x$$,
(ii) $$x\le y$$ implies $$y'\le x'$$

for all $$x,y\in L$$.

It is worth noticing that a Sheffer operation need not be unique, see Lemma 2.3.

The following lemma was shown for ortholattices in Chajda (2005), but it holds also for lattices with antitone involution.

### Lemma 2.3

Let $(L,\vee ,\wedge ,{}')$ be a lattice with antitone involution. Then, (i) and (ii) hold:

(i) If $x|y:=x'\vee y'$ for all $x,y\in L$, then (*L*, |) is a Sheffer groupoid.
(ii) If $x|y:=x'\wedge y'$ for all $x,y\in L$, then (*L*, |) is a Sheffer groupoid.

### Proof

(i) Since $x|x\approx x'\vee x'\approx x'$, (1) and (2) are equivalent to
  $$\begin{aligned} (x'\vee y')'\vee x''&\approx x, \\ (x'\vee y')'\vee y''&\approx y, \end{aligned}$$
  respectively.
(ii) Since $x|x\approx x'\wedge x'\approx x'$, (1) and (2) are equivalent to
  $$\begin{aligned} (x'\wedge y')'\wedge x''&\approx x, \\ (x'\wedge y')'\wedge y''&\approx y, \end{aligned}$$
  respectively.

$$\square $$

The question whether one of the identities (1) and (2) implies the other one is answered in the negative by the following lemma.

### Lemma 2.4

Axioms (1) and (2) are independent.

### Proof

If $A:=\{a,b\}$ and the binary operation | on *A* is defined by $x|y:=x$ for all $x,y\in A$, then | satisfies (1), but not (2) since $(a|b)|(b|b)=a|b=a\ne b$. If $A:=\{a,b,c\}$ and the binary operation | on *A* is defined by

$$\begin{aligned} \begin{array}{c|ccc} | &{}\quad a &{}\quad b &{}\quad c \\ \hline a &{}\quad a &{}\quad b &{}\quad c \\ b &{}\quad c &{}\quad b &{}\quad c \\ c &{}\quad a &{}\quad a &{}\quad c, \end{array} \end{aligned}$$

then | satisfies (2), but not (1) since $(a|b)|(a|a)=b|a=c\ne a$. $\square$

Let us recall some concepts from theory of relations.

Let *A* be a non-void set, $a,b\in A$, *R* a binary relation on *A* and $'$ a unary operation on *A*. We define

$$\begin{aligned} U(a,b)&:=\{x\in A\mid (a,x),(b,x)\in R\}, \\ L(a,b)&:=\{x\in A\mid (x,a),(x,b)\in R\} \end{aligned}$$

and call these sets the *upper cone* and *lower cone* of *a* and *b* with respect to *R*, respectively. The relational system ${\mathbf {A}}=(A,R)$ is called *directed* if $U(x,y)\ne \emptyset$ and $L(x,y)\ne \emptyset$ for all $x,y\in A$. The operation $'$ is called *antitone* if $(x,y)\in R$ implies $(y',x')\in R$ and an *involution* on ${\mathbf {A}}$ if it is antitone and if it satisfies the identity $x''\approx x$. The relational system (*B*, *S*) is called a *subsystem* of ${\mathbf {A}}$ if $B\subseteq A$ and $S=R\cap B^2$.

### Lemma 2.5

Let $(A,R,{}')$ be a relational system with involution and assume $U(x,y)\ne \emptyset$ for all $x,y\in A$. Then, (*A*, *R*) is directed.

### Proof

We have $L(x,y)\ne \emptyset$ for all $x,y\in A$ since $L(x,y)\approx (U(x',y'))'$, where $B':=\{b'\mid b\in B\}$ for every subset *B* of *A*. $\square$

### Definition 2.6

A *directed relational system with involution* is an ordered triple $(A,R,{}')$ consisting of a non-void set *A*, a binary relation *R* on *A* and a unary operation $'$ on *A* satisfying the following conditions:

3 $$\begin{aligned}&R\text { is reflexive}, \end{aligned}$$

4 $$\begin{aligned}&(A,R)\text { is directed}, \end{aligned}$$

5 $$\begin{aligned}&'\text { is an involution on }(A,R). \end{aligned}$$

## Representation of relational systems by Sheffer groupoids

The following result shows how a Sheffer groupoid is connected with a directed relational system with involution.

For every Sheffer groupoid ${\mathbf {A}}=(A,|)$ put ${\mathbb {R}}({\mathbf {A}}):=(A,R,{}')$ where

$$\begin{aligned} x'&:=x|x\text { for all }x\in A, \\ R&:=\{(x,y)\in A^2\mid x'|y'=y\}. \end{aligned}$$

### Theorem 3.1

Let ${\mathbf {A}}=(A,|)$ be a Sheffer groupoid. Then, ${\mathbb {R}}({\mathbf {A}})$ is a directed relational system with involution, the so-called *directed relational system with involution induced by* ${\mathbf {A}}$.

### Proof

Let $a,b\in A$. (1) implies $x''\approx x$ and that *R* is reflexive. (1) and (2) can be written in the equivalent form $(x|y)|x'\approx x$ and $(x|y)|y'\approx y$, respectively. If $(a,b)\in R$, then $a'|b'=b$, and hence $b|a=(a'|b')|a=a'$, i.e., $(b',a')\in R$ showing that $'$ is an involution on (*A*, *R*). Since $(a'|b')|a=a'$ and $(a'|b')|b=b'$, we have $((a'|b')',a'),((a'|b')',b')\in R$, and hence $(a,a'|b'),(b,a'|b')\in R$, i.e., $a'|b'\in U(a,b)$ which shows $U(a,b)\ne \emptyset$. According to Lemma 2.5, (*A*, *R*) is directed. $\square$

### Example 3.2

Put $A:=\{a,b,c,d\}$ and define a unary operation $'$ on *A* by

$$\begin{aligned} \begin{array}{l|llll} x &{} a &{} b &{} c &{} d \\ x' &{} a &{} b &{} d &{} c \end{array} \end{aligned}$$

Then, $(A,A^2\setminus \{(a,b),(b,a)\},{}')$ is the directed relational system induced by the Sheffer groupoid ${\mathbf {A}}$ from Example 2.2.

In the following, we show that also conversely, to every directed relational system with involution, a Sheffer groupoid can be assigned.

Let ${\mathbf {A}}=(A,R,{}')$ be a directed relational system with involution. Define a binary operation | on *A* as follows: if $(x',y')\in R$, then $x|y:=y'$, and take *x*|*y* as an arbitrary element of $U(x',y')$, otherwise ($x,y\in A$). Then, | will be called an *operation assigned to* ${\mathbf {A}}$.

### Lemma 3.3

Let ${\mathbf {A}}=(A,R,{}')$ be a directed relational system with involution and | a binary operation on *A*. Then, | is assigned to ${\mathbf {A}}$ if and only if

(i) $(x,y)\in R$ if and only if $x'|y'=y$,
(ii) $x|y\in U(x',y')$ for all $x,y\in A$.

### Proof

Let $a,b\in A$. First assume | to be assigned to ${\mathbf {A}}$. If $(a,b)\in R$, then $(a'',b'')\in R$, and hence $a'|b'=b''=b$. Conversely, assume $a'|b'=b$. Then, $(a,b)\notin R$ would imply $(a'',b'')\notin R$, and hence $b=a'|b'\in U(a'',b'')=U(a,b)$ and hence $(a,b)\in R$, a contradiction. Hence, $(a,b)\in R$. This shows (i). If $(a',b')\in R$, then $a|b=b'\in U(a',b')$. Otherwise, $a|b\in U(a',b')$, too. This shows (ii). Conversely, if | satisfies (i) and (ii), then clearly | is assigned to ${\mathbf {A}}$. $\square$

The following lemma follows easily.

### Lemma 3.4

If $(A,R,{}')$ is a directed relational system with involution and | an assigned operation, then condition (ii) of Lemma 3.3 is equivalent to

$$\begin{aligned} (x|y)|(x|y)\in L(x,y)\text { for all }x,y\in A. \end{aligned}$$

In the following, we will often use this lemma. Now, we prove the converse of Theorem 3.1.

Let ${\mathbf {A}}=(A,R,{}')$ be a directed relational system with involution. Then, ${\mathbb {G}}({\mathbf {A}}):=(A,|)$, where | is an operation assigned to ${\mathbf {A}}$.

### Theorem 3.5

Let ${\mathbf {A}}=(A,R,{}')$ be a directed relational system with involution. Then, ${\mathbb {G}}({\mathbf {A}})=(A,|)$ is a Sheffer groupoid, a so-called *Sheffer groupoid assigned to* ${\mathbf {A}}$, and | is called a *Sheffer operation assigned to *${\mathbf {A}}$.

### Proof

Let ${\mathbb {G}}({\mathbf {A}})=(A,|)$. We show that (*A*, |) satisfies equations (1) and (2) and hence is a Sheffer groupoid. Let $a,b\in A$. Since $(x',x')\in R$, we have $x|x\approx x'$. If $(a',b')\in R$, then $(b,a)\in R$, and hence $(a|b)|a'=b'|a'=a$ and $(a|b)|b'=b'|b'=b$. If $(a',b')\notin R$, then $a|b\in U(a',b')$, and hence $(a',a|b),(b',a|b)\in R$ which implies $((a|b)',a),((a|b)',b)\in R$, i.e., $(a|b)|a'=a$ and $(a|b)|b'=b$. $\square$

### Remark 3.6

In general, ${\mathbb {G}}({\mathbf {A}})$ is not uniquely defined. However, it contains all the information on the directed relational system ${\mathbf {A}}$ with involution. In other words, the given directed relational system with involution can be completely recovered from an assigned Sheffer groupoid, see the following result.

### Theorem 3.7

Let ${\mathbf {A}}=(A,R,{}')$ be a directed relational system with involution. Then, ${\mathbb {R}}({\mathbb {G}}({\mathbf {A}}))={\mathbf {A}}$.

### Proof

If

$$\begin{aligned} {\mathbb {G}}({\mathbf {A}})&=(A,|), \\ {\mathbb {R}}({\mathbb {G}}({\mathbf {A}}))&=(A,S,{}^*), \end{aligned}$$

then according to Lemma 3.3,

$$\begin{aligned} S&=\{(x,y)\in A^2\mid x'|y'=y\}\\&=\{(x,y)\in A^2\mid (x,y)\in R\}=R, \\ x^*&\approx x|x\approx x'. \qquad \qquad \qquad \qquad \qquad \qquad \qquad \qquad \qquad \qquad \qquad \square \end{aligned}$$

On the other hand, we can show for which pairs of elements a Sheffer operation $\circ$ assigned to ${\mathbb {R}}(A,|)$ coincides with the Sheffer operation | of a given Sheffer groupoid (*A*, |).

### Theorem 3.8

Let ${\mathbf {A}}=(A,|)$ be a Sheffer groupoid and ${\mathbb {G}}({\mathbb {R}}({\mathbf {A}}))=(A,\circ )$. Then, $x\circ y=x|y$ if $x|y=y|y$.

### Proof

If ${\mathbb {R}}({\mathbf {A}})=(A,R,{}')$, then any of the following assertions implies the next one:

$$\begin{aligned} x|y&=y|y, \\ x|y&=y', \\ (x',y')&\in R, \\ x\circ y&=y', \\ x\circ y&=x|y. \end{aligned}$$

$$\square $$

In fact, $\circ$ need not coincide with | as can be seen by the following example.

### Example 3.9

If | is the Sheffer operation from Example 2.2, then $\circ$ has the operation table

$$\begin{aligned} \begin{array}{c|cccc} \circ &{}\quad a &{}\quad b &{}\quad c &{}\quad d \\ \hline a &{}\quad a &{}\quad x &{}\quad d &{}\quad c \\ b &{}\quad y &{}\quad b &{}\quad d &{}\quad c \\ c &{}\quad a &{}\quad b &{}\quad d &{}\quad c \\ d &{}\quad a &{}\quad b &{}\quad d &{}\quad c, \end{array} \end{aligned}$$

where $x,y\in \{c,d\}$ since $U(a,b)=\{c,d\}$ in the induced relational system. Hence, if we take $x=d$ or $y=d$, then $\circ$ differs from |.

Theorem 3.7 shows that if we start with a directed relational system ${\mathbf {A}}$ with involution, we consider a Sheffer groupoid ${\mathbf {G}}$ assigned to ${\mathbf {A}}$, and we construct the directed relational system ${\mathbf {B}}$ with involution induced by ${\mathbf {G}}$, then ${\mathbf {B}}={\mathbf {A}}$. Hence, the Sheffer operation substitutes both the binary relation and the unary operation analogously to the situation for Boolean algebras where the Sheffer operation substitutes all other fundamental operations of the Boolean algebra.

## Elementary properties of relations

In the following, we characterize properties of the relation *R* of a directed relational system ${\mathbf {A}}=(A,R,{}')$ with involution by means of identities and quasi-identities for a Sheffer operation assigned to ${\mathbf {A}}$.

### Theorem 4.1

Let ${\mathbf {A}}=(A,R,{}')$ be a directed relational system with involution and | an assigned Sheffer operation. Then, *R* is symmetric if and only if | satisfies the identity

6 $$\begin{aligned} ((x|y)|(x|y))|x\approx x|x. \end{aligned}$$

### Proof

If *R* is symmetric, then any of the following assertions implies the next one:

$$\begin{aligned}&x|y \in U(x',y'), \\&\quad (x',x|y) \in R, \\&\quad (x|y,x') \in R, \\&\quad (x|y)'|x \approx x', \\&\quad ((x|y)|(x|y))|x \approx x|x. \end{aligned}$$

If, conversely, | satisfies identity (6), then any of the following assertions implies the next one:

$$\begin{aligned}&(x,y) \in R, \\&\quad x'|y' =y, \\&\quad y'|x' =(x'|y')'|x'=x, \\&\quad (y,x) \in R. \end{aligned}$$

$$\square $$

Another important property of a binary relation is antisymmetry. Recall that a binary relation *R* is antisymmetric if $(x,y),(y,x)\in R$ implies $x=y$.

### Theorem 4.2

Let ${\mathbf {A}}=(A,R,{}')$ be a directed relational system with involution and | a Sheffer operation assigned to it. Then, the following holds:

(i) *R* is antisymmetric if and only if $x|y=y'$ and $y|x=x'$ imply $x=y$.
(ii) If $x|y\approx y|x$, then *R* is antisymmetric.

### Proof

(i) Immediate by (i) of Lemma 3.3.
(ii) This follows from (i), since $x|y\approx y|x$, $x|y=y'$ and $y|x=x'$ imply $x=(y|x)'=(x|y)'=y$.

$$\square $$

Transitivity of a binary relation can be expressed by an identity for an assigned Sheffer operation as follows.

### Theorem 4.3

Let ${\mathbf {A}}=(A,R,{}')$ be a directed relational system with involution and | an assigned Sheffer operation. Then, *R* is transitive if and only if | satisfies the identity

7 $$\begin{aligned} x|(((((x|y)|(x|y))|z)|(((x|y)|(x|y))|z))\approx ((x|y)|(x|y))|z.\nonumber \\ \end{aligned}$$

### Proof

If *R* is transitive, then any of the following assertions implies the next one:

$$\begin{aligned} x|y\in U(x',y')&\text { and }(x|y)'|z\in U(x|y,z'), \\ (x',x|y),(x|y,(x|y)'|z)&\in R, \\ (x',(x|y)'|z)&\in R, \\ x|((x|y)'|z)'&\approx (x|y)'|z, \\ x|(((((x|y)|(x|y))|z)|(((x|y)|(x|y))|z))&\approx ((x|y)|(x|y))|z. \end{aligned}$$

If, conversely, | satisfies identity (7), then any of the following assertions implies the next one:

$$\begin{aligned} (x,y),(y,z)&\in R, \\ x'|y'=y&\text { and }y'|z'=z, \\ x'|z'&=x'|(y'|z')'=x'|((x'|y')'|z')'\\&=(x'|y')'|z'=y'|z'=z, \\ (x,z)&\in R. \end{aligned}$$

$$\square $$

Let us introduce the following concepts. A *bounded relational system with involution* is an ordered quintuple ${\mathbf {A}}=(A,R,{}',$ 0, 1) such that $(A,R,{}')$ is a directed relational system with involution, $0,1\in A$ and $(0,x),(x,1)\in R$ hold for all $x\in A$. ${\mathbf {A}}$ is called *complemented* if it is bounded and if $U(x,x')\approx 1\approx 0'$. In such a case, $L(x,x')\approx 0$. Also these properties of relational systems can be characterized by identities and quasi-identities for an assigned Sheffer operation.

### Theorem 4.4

Let $(A,R,{}')$ be a directed relational system with involution and | a Sheffer operation assigned to it. Moreover, let $0,1\in A$ and put ${\mathbf {A}}:=(A,R,{}',0,1)$. Then, the following holds:

(i) ${\mathbf {A}}$ is bounded if and only if it satisfies the identities $(0|0)|x\approx x|x$ and $x|(1|1)\approx 1$.
(ii) ${\mathbf {A}}$ is complemented if it is bounded, $0|0\approx 1$ and if for every $x,y\in A$,
  $$\begin{aligned} x|(y|y)=(x|x)|(y|y)=y\text { implies }y=1. \end{aligned}$$

### Proof

(i) The assertions $(0,x')\in R$ and $(x',1)\in R$ are equivalent to $0'|x\approx x'$ and $x|1'\approx 1$, respectively.
(ii) The following are equivalent:
  $$\begin{aligned} y&\in U(x,x'), \\ (x,y),(x',y)&\in R, \\ x|y'&=x'|y'=y, \\ x|(y|y)&=(x|x)|(y|y)=y. \end{aligned}$$

$$\square $$

As mentioned in Section 2, the class of Sheffer groupoids forms a variety ${\mathcal {S}}$. We can ask one more condition, namely commutativity of |. As shown in Theorems 3.7 and 4.2, the directed relational systems with involution induced by commutative Sheffer groupoids will have antisymmetric binary relations. We present a subvariety of the variety ${\mathcal {S}}$ containing all commutative Sheffer groupoids which has an important congruence property.

We recall that a variety ${\mathcal {V}}$ of algebras is called *congruence distributive* if every member of ${\mathcal {V}}$ has a distributive congruence lattice.

### Theorem 4.5

The variety ${\mathcal {T}}$ of Sheffer groupoids (*A*, |) satisfying the identities

8 $$\begin{aligned}&(x|y)|(x|x)\approx (x|x)|(x|y), \end{aligned}$$

9 $$\begin{aligned}&(x|y)|(y|y)\approx (y|y)|(x|y) \end{aligned}$$

is congruence distributive.

### Proof

We show that the variety ${\mathcal {T}}$ possesses a majority term, i.e., a ternary term *m* satisfying

$$\begin{aligned} m(x,x,y)\approx m(x,y,x)\approx m(y,x,x)\approx x \end{aligned}$$

which implies the congruence distributivity of ${\mathcal {T}}$. If

$$\begin{aligned} x':=x|x\text { and }m(x,y,z):=((x|y)|(x|z))'|(y|z), \end{aligned}$$

then

$$\begin{aligned} m(x,x,y)&\approx ((x|x)|(x|y))'|(x|y)\approx x'|(x|y)\approx x\text { by (8) }\\&\text {and (1)}, \\ m(x,y,x)&\approx ((x|y)|(x|x))'|(y|x)\approx x'|(y|x)\approx x\text { by (1),}\\&\text {(9) and (2)}, \\ m(y,x,x)&\approx ((y|x)|(y|x))'|(x|x)\approx (y|x)|x'\approx x\text { by (2)}. \end{aligned}$$

$$\square $$

## Kleene relational systems and twist products

At first, we show how homomorphisms of Sheffer groupoids are related with homomorphisms of induced directed relational systems with involution. Because in the literature there are different concepts of homomorphism of relational systems, we recall the following one.

Let (*A*, *R*) and (*B*, *S*) be relational systems. A mapping $f:A\rightarrow B$ is called a *homomorphism* from (*A*, *R*) to (*B*, *S*) if

$$\begin{aligned} (x,y)\in R\text { implies }(f(x),f(y))\in S. \end{aligned}$$

A *homomorphism* f is called *strong* if

$$\begin{aligned} (x,y)\in R\text { if and only if }(f(x),f(y))\in S. \end{aligned}$$

If $(A,R,{}')$ and $(B,S,^*)$ are relational systems with unary operation, then *f* is a *homomorphism* from $(A,R,{}')$ to $(B,S,^*)$ if it is a homomorphism from (*A*, *R*) to (*B*, *S*) satisfying

$$\begin{aligned} f(x')=(f(x))^*\text { for all }x\in A. \end{aligned}$$

### Theorem 5.1

Let ${\mathbf {A}}=(A,|_A)$ and ${\mathbf {B}}=(B,|_B)$ be Sheffer groupoids and *f* a homomorphism from ${\mathbf {A}}$ to ${\mathbf {B}}$. Then, *f* is a homomorphism between the induced directed relational systems ${\mathbb {R}}({\mathbf {A}})$ and ${\mathbb {R}}({\mathbf {B}})$ with involution.

### Proof

Let $a,b\in A$, ${\mathbb {R}}({\mathbf {A}})=(A,R,{}')$ and ${\mathbb {R}}({\mathbf {B}})=(B,S,{}^*)$. We have $f(x')\approx f(x|_Ax)\approx f(x)|_Bf(x)\approx (f(x))^*$ and hence any of the following assertions implies the next one:

$$\begin{aligned} (a,b)&\in R, \\ a'|_Ab'&=b, \\ f(a'|_Ab')&=f(b), \\ f(a')|_Bf(b')&=f(b), \\ (f(a))^*|_B(f(b))^*&=f(b), \\ (f(a),f(b))&\in S. \end{aligned}$$

$$\square $$

For the converse direction, we firstly mention the following result for bounded relational systems.

### Lemma 5.2

Let $(A,R,{}',0_A,1_A)$ and $(B,S,^*,0_B,1_B)$ be bounded relational systems with involution and *f* a strong homomorphism from ${\mathbf {A}}=(A,R,{}')$ to ${\mathbf {B}}=(B,S,^*)$. Further assume that $f(1_A)=1_B$. Define binary operations $|_A$ and $|_B$ on *A* and *B*, respectively, by

$$\begin{aligned} x|_Ay:=\left\{ \begin{array}{ll} y' &{} \text {if }(x',y')\in R, \\ 1_A &{} \text {otherwise} \end{array} \right. \quad x|_By:=\left\{ \begin{array}{ll} y^* &{} \text {if }(x^*,y^*)\in S, \\ 1_B &{} \text {otherwise} \end{array} \right. \end{aligned}$$

Then, $(A,|_A)$ and $(B,|_B)$ are Sheffer groupoids assigned to ${\mathbf {A}}$ and ${\mathbf {B}}$, respectively, and *f* is a homomorphism from $(A,|_A)$ to $(B,|_B)$.

### Proof

Let $a,b\in A$. Obviously, $(A,|_A)$ and $(B,|_B)$ are Sheffer groupoids. If $(a',b')\in R$, then $((f(a))^*,(f(b))^*)=(f(a'),f(b'))\in S$, and hence $f(a|_Ab)=f(b')=(f(b))^*=f(a)|_Bf(b)$. If $(a',b')\notin R$, then $((f(a))^*,(f(b))^*)=(f(a'),f(b'))\notin S$, and hence $f(a|_Ab)=f(1_A)=1_B=f(a)|_Bf(b)$. $\square$

We are going to determine conditions under which the converse of Theorem 5.1 holds.

### Theorem 5.3

Let ${\mathbf {A}}=(A,R,{}')$ and ${\mathbf {B}}=(B,S,{}^*)$ be directed relational systems with involution, *f* a strong surjective homomorphism from ${\mathbf {A}}$ to ${\mathbf {B}}$ and $|_A$ a Sheffer operation assigned to ${\mathbf {A}}$. Further, assume that the equivalence relation $\ker f$ on *A* is a congruence on $(A,|_A)$. Then, there exists a Sheffer operation $|_B$ on *B* such that *f* is a homomorphism from $(A,|_A)$ to $(B,|_B)$ and $|_B$ is assigned to ${\mathbf {B}}$.

### Proof

Define $f(x)|_Bf(y):=f(x|_Ay)$ for all $x,y\in A$. Since $\ker f\in {{\,\mathrm{Con}\,}}(A,|_A)$ and *f* is surjective, $|_B$ is well-defined. Let $a,b\in A$. Then, any of the following assertions implies the next one:

$$\begin{aligned} ((f(a))^*,(f(b))^*)&\in S, \\ (f(a'),f(b'))&\in S, \\ (a',b')&\in R, \\ a|_Ab&=b', \\ f(a|_Ab)&= f(b'), \\ f(a)|_Bf(b)&=(f(b))^*. \end{aligned}$$

Moreover, any of the following assertions implies the next one:

$$\begin{aligned} ((f(a))^*,(f(b))^*)&\notin S, \\ (f(a'),f(b'))&\notin S, \\ (a',b')&\notin R, \\ a|_Ab&\in U(a',b'), \\ f(a|_Ab)&\in U(f(a'),f(b')), \\ f(a)|_Bf(b)&\in U((f(a))^*,(f(b))^*). \end{aligned}$$

This shows that $|_B$ is a Sheffer operation on *B* assigned to ${\mathbf {B}}$. According to the definition of $|_B$, we have $f(x|_Ay)=f(x)|_Bf(y)$ for all $x,y\in A$, whence *f* is a homomorphism from $(A,|_A)$ to $(B,|_B)$. The proof is settled. $\square$

For a lattice ${\mathbf {L}}=(L,\vee ,\wedge )$, its twist product $(L^2,\sqcup ,\sqcap )$ is defined by

$$\begin{aligned} (x,y)\sqcup (z,v)&:=(x\vee z,v\wedge y), \\ (x,y)\sqcap (z,v)&:=(x\wedge z,v\vee y) \end{aligned}$$

for all $(x,y),(z,v)\in L^2$. We extend this concept to relational systems as follows.

Let *A* be a non-void set and *R* a binary relation on *A*. Then, $(A^2,S,{}^*)$ with

$$\begin{aligned} S&:=\{((x,y),(z,v))\in (A^2)^2\mid (x,z),(v,y)\in R\}, \\ (x,y)^*&:=(y,x) \end{aligned}$$

for all $(x,y)\in A^2$ will be called the *twist product of (A, R)*.

Recall that an *embedding* of a relational system ${\mathbf {A}}$ into a relational system ${\mathbf {B}}$ is an injective strong homomorphism from ${\mathbf {A}}$ to ${\mathbf {B}}$.

The importance of twist products is pointed out by the next result.

### Theorem 5.4

Let ${\mathbf {A}}=(A,R)$ be a relational system, $a\in A$ and ${\mathbf {B}}=(A^2,S,{}^*)$, the twist product of ${\mathbf {A}}$. Then, the following holds:

(i) If ${\mathbf {A}}$ is directed, then ${\mathbf {B}}$ is a directed relational system with involution $^*$,
(ii) the mapping $x\mapsto (x,a)$ is an embedding of ${\mathbf {A}}$ into $(A^2,S)$.

### Proof

Let $$a,b,c,d\in A$$.

(i) Assume $${\mathbf {A}}$$ to be directed. Because of $$U((a,b),(c,d))=U(a,c)\times L(b,d)$$, $$(A^2,S)$$ is directed. Moreover, $$(x,y)^{**}\approx (y,x)^*\approx (x,y)$$, and the following are equivalent:
  $$\begin{aligned} ((a,b),(c,d))&\in S, \\ (a,c),(d,b)&\in R, \\ (d,b),(a,c)&\in R, \\ ((d,c),(b,a))&\in S, \\ ((c,d)^*,(a,b)^*)&\in S. \end{aligned}$$
  Hence, $^*$ is an involution on $(A^2,S)$.
(ii) The mapping $x\mapsto (x,a)$ is injective. Moreover, $((b,a),(c,a))\in S$ if and only if $(b,c)\in R$. $\square$

Hence, every directed relational system can be embedded into a directed relational system with involution.

The question arises whether a Sheffer operation assigned to the twist product of a directed relational system ${\mathbf {A}}$ with involution can be derived from a Sheffer operation assigned to ${\mathbf {A}}$. We give a positive answer in the following theorem.

### Theorem 5.5

Let $(A,R,{}')$ be a directed relational system with involution, $|_A$ an assigned Sheffer operation on *A* and define

$$\begin{aligned} (x,y)|_B(z,v):=(y'|_Av',(x|_Az)') \end{aligned}$$

for all $(x,y),(z,v)\in A^2$. Then, $|_B$ is a Sheffer operation on $A^2$ assigned to the twist product of (*A*, *R*).

### Proof

For $a,b,c,d\in A$, the following are equivalent:

$$\begin{aligned} ((a,b)^*,(c,d)^*)&\in S, \\ ((b,a),(d,c))&\in S, \\ (b,d),(c,a)&\in R, \\ (b'',d''),(a',c')&\in R, \\ (b'|_Ad',a|_Ac)&=(d'',c'), \\ (b'|_Ad',(a|_Ac)')&=(d,c), \\ (a,b)|_B(c,d)&=(d,c), \\ (a,b)|_B(c,d)&=(c,d)^* \end{aligned}$$

and the following are equivalent:

$$\begin{aligned} (b'|_Ad',a|_Ac)&\in U(b'',d'')\times U(a',c'), \\ (b'|_Ad',(a|_Ac)')&\in U(b,d)\times L(a,c), \\ (b'|_Ad',(a|_Ac)')&\in U((b,a),(d,c)), \\ (a,b)|_B(c,d)&\in U((a,b)^*,(c,d)^*). \end{aligned}$$

$$\square $$

In order to simplify notation, we extend binary relations between elements of a non-void set *A* to relations between subsets of *A*.

Let *A* be a non-void set, *b*, *c* be elements of *A*, *B*, *C* be subsets of *A* and *R* be a binary relation on *A*. We say $(B,C)\in R$ if $B\times C\subseteq R$. Instead of $(\{b\},C)\in R$ and $(B,\{c\})\in R$, we shortly write $(b,C)\in R$ and $(B,c)\in R$, respectively.

The concept of a Kleene lattice was introduced by J. A. Kalman in Kalman (1958). Recall that a distributive lattice $(L,\vee ,\wedge ,{}')$ with antitone involution is called a *Kleene lattice* if it satisfies the so-called *normality condition*, i.e. the identity

$$\begin{aligned} x\wedge x'\le y\vee y'\text { for all }x,y\in L. \end{aligned}$$

These lattices are used in logic in order to formalize certain De Morgan propositional logics. For posets with involution, this notion was already generalized by Chajda and Länger (to appear in Miskolc Math Notes), in the following way. A distributive poset $(P,\le ,{}')$ with involution is called a *Kleene poset* if

$$\begin{aligned} L(x,x')\le U(y,y')\text { for all }x,y\in P \end{aligned}$$

which means that $z\le v$ for all $x,y\in P$ and all $(z,v)\in L(x,x')\times U(y,y')$.

### Definition 5.6

(i) A *Kleene relational system* is a relational system $(A,R,{}')$ with antitone involution satisfying
  $$\begin{aligned} (L(x,x'),U(y,y'))\in R\text { for all }x,y\in A. \end{aligned}$$
(ii) If $${\mathbf {A}}=(A,R)$$ is a relational system, $$a\in A$$ and $$(A^2,S,$$ $${}^*)$$ the twist product of $${\mathbf {A}}$$, then we define the following subset of $$A^2$$:
  $$\begin{aligned} P_a({\mathbf {A}}):=\{(x,y)\in A^2\mid (L(x,y),a),(a,U(x,y))\in R\}. \end{aligned}$$

It is worth noticing that Kleene lattices and Kleene posets are Kleene relational systems according to our previous definition.

Using the above-defined subset of the twist product, we can show that every directed relational system with a transitive relation can be embedded into a Kleene relational system.

### Theorem 5.7

Let ${\mathbf {A}}=(A,R)$ be a relational system, $a\in A$, $(A^2,S,{}^*)$ the twist product of ${\mathbf {A}}$ and $T:=S\cap (P_a({\mathbf {A}}))^2$. Then, the following holds:

(i) If *R* is transitive then $(P_a({\mathbf {A}}),T,{}^*)$ is a relational system with involution which is a Kleene relational system,
(ii) the mapping $x\mapsto (x,a)$ is an embedding of ${\mathbf {A}}$ into $(P_a({\mathbf {A}}),T)$.

### Proof

Let $$(b,c),(d,e)\in P_a({\mathbf {A}})$$.

(i) Put ${\mathbf {B}}:=(P_a({\mathbf {A}}),T,{}^*)$. From $(b,c)\in P_a({\mathbf {A}})$, we conclude $(L(b,c),a),(a,U(b,c))\in R$ and hence $(L(c,b),a),(a,U(c,b))\in R$, i.e. $(b,c)^*=(c,b)\in P_a({\mathbf {A}})$ which shows that $P_a({\mathbf {A}})$ is closed with respect to $^*$. Because of
  $$\begin{aligned} (L((b,c),(c,b)),(a,a))=(L(b,c)\times U(b,c),(a,a))&\in S, \\ ((a,a),U(d,e)\times L(d,e))=((a,a),U((d,e),(e,d)))&\in S, \end{aligned}$$
  we have $(L((b,c),(c,b)),U((d,e),(e,d)))\in S$ due to transitivity of *S* (which follows from the transitivity of *R*), and hence ${\mathbf {B}}$ is a Kleene relational system.
(ii) For all $x\in A$, we have $(L(x,a),a),(a,U(x,a))\in R$, and hence $(x,a)\in P_a({\mathbf {A}})$. The rest follows from Theorem 5.4. $\square$

It should be remarked that if *R* is transitive, then $(P_a({\mathbf {A}}),T,{}^*)$ is a relational subsystem of the twist product $(A^2,S,{}^*)$ of ${\mathbf {A}}$.

## Conclusion

We have shown that to every directed relational system ${\mathbf {A}}=(A,R,{}')$ with involution, there can be assigned a Sheffer operation | on *A* such that the given system ${\mathbf {A}}$ can be reconstructed from the groupoid (*A*, |). Hence, investigations of these relational systems can be transferred to Sheffer groupoids, i.e., algebras forming a variety ${\mathcal {S}}$ defined by two simple identities which contains a non-trivial congruence distributive subvariety. Hence, properties of the binary relation *R* can be characterized by means of identities and quasi-identities of the assigned Sheffer groupoid. This is important because tools of general algebra are more developed than those of the theory of relations. Moreover, we have shown how every relational system (*A*, *R*) can be converted into a Kleene relational system by using the twist product construction. Remember that Kleene relational systems play an important role in substructural logics. If the Kleene relational system is constructed from a relational system with involution, then we showed how the Sheffer operation can be constructed. This research should go on in a purely algebraic way since we can study, e.g., subdirectly irreducible members or free algebras of the variety ${\mathcal {S}}$ by using the famous Jónsson’s Lemma and the corresponding relational systems. However, these are topics for future research.
